# Inhibition of epidermal growth factor signaling by the cardiac glycoside ouabain in medulloblastoma

**DOI:** 10.1002/cam4.314

**Published:** 2014-07-23

**Authors:** Daniel Wolle, Seung Joon Lee, Zhiqin Li, Alisa Litan, Sonali P Barwe, Sigrid A Langhans

**Affiliations:** Nemours Center for Childhood Cancer Research, Alfred I. duPont Hospital for ChildrenWilmington, Delaware, 19803

**Keywords:** Cardiac glycosides, epidermal growth factor, medulloblastoma, Na,K-ATPase, ouabain

## Abstract

Epidermal growth factor (EGF) signaling regulates cell growth, proliferation, and differentiation. Upon receptor binding, EGF triggers cascades of downstream signaling, including the MAPK and phosphoinositide-3-kinase (PI3K)/Akt signaling pathways. Aberrant expression/activation of EGFR is found in multiple human cancers, including medulloblastoma, the most prevalent pediatric brain cancer, and often has been associated with metastasis, poor prognosis, and resistance to chemotherapy. Na,K-ATPase is an ion pump well known for its role in intracellular ion homeostasis. Recent studies showed that Na,K-ATPase also functions as a signaling platform and revealed a role in EGFR, MAPK, and PI3K signaling. While both EGFR and Na,K-ATPase seem to modulate similar signaling pathways, cardiac glycosides that are steroid-like inhibitors of Na,K-ATPase, exhibit antiproliferative and proapoptotic properties in cancer cells. Thus, we sought to better understand the relationship between EGF and cardiac glycoside signaling. Here, we show that in medulloblastoma cells, both EGF and ouabain activate Erk1/2 and PI3K/Akt signaling. Nevertheless, in medulloblastoma cells ouabain did not transactivate EGFR as has been reported in various other cell lines. Indeed, ouabain inhibited EGF-induced Erk1/2 and Akt activation and, moreover, prevented EGF-induced formation of actin stress fibers and cell motility, probably by activating a stress signaling response. Na,K-ATPase has been proposed to act as a signaling scaffold and our studies suggest that in medulloblastoma cells Na,K-ATPase might act as a check point to integrate EGF-associated signaling pathways. Thus, Na,K-ATPase might serve as a valid target to develop novel therapeutic approaches in tumors with aberrant activation of the EGFR signaling cascades.

## Introduction

Medulloblastoma, which usually arises in the cerebellum, is the most common malignant form of brain cancer in children 1. Despite aggressive treatment regimens and recent improvements in survival rates, medulloblastoma is still associated with substantial mortality and patients that survive often suffer from serious lifelong therapy-related side effects 1. Conventionally, medulloblastoma patients were stratified into high- and average-risk groups based on clinical parameters such as patient age, extent of disease at the time of diagnosis, and completeness of surgical resection. In recent years, much progress has been made in molecular stratification of medulloblastoma patients to predict disease outcome and develop targeted therapies. Now several subtypes with distinct developmental origins, genetic profiles, pathway signatures, and clinicopathological features have been identified. The current consensus divides medulloblastoma into four distinct subgroups, WNT, sonic hedgehog (SHH), Group 3 and Group 4 allowing for different targeted therapeutic approaches 1. While not specifically restricted to a defined subgroup, aberrant activation of epidermal growth factor receptor (EGFR) signaling has been associated with poor outcome 2 and targeting of the EGFR signaling cascade might be of additional therapeutic value 3–5.

The EGFR family of receptor tyrosine kinases regulates various cellular functions as diverse as growth, differentiation, cell motility, and survival. Abnormal expression, activation, and mutation of EGFR family members have all been implicated in the progression of various cancers. The EGFR family consists of four members, EGFR/ErbB1, HER2/ErbB2, HER3/ErbB3, and HER4/ErbB4. Upon ligand binding, the receptors form homo- or heterodimers thereby activating the intracellular tyrosine kinase domain and triggering various downstream signaling cascades. These include the Erk1/2 (MAPK) and phosphoinositide-3 (PI3)-kinase/Akt pathways which are known to play fundamental roles in cellular proliferation, survival, and differentiation in fibroblasts and epithelial cells. While EGFR is expressed in glial cells and neurons of the hippocampus, cerebral cortex, and cerebellum, the role of EGFR signaling in the central nervous system (CNS) is less well characterized. EGF responses in the CNS range from proliferation and survival to maturation of neurons and neuronal precursors 6,7. In cerebellar granule neurons, EGFR activation has antineuritogenic properties and inhibits neurite outgrowth 8. In medulloblastoma, which is thought to arise from cerebellar granule cell precursors, ErbB2 overexpression has been associated with advanced metastatic disease and poor clinical outcome 2. ErbB2 is expressed in more than 80% of tumors and upregulates prometastatic genes and increases the migration of medulloblastoma cells 2,9,10. Thus, aberrant EGFR/ErbB2 signaling has become an attractive therapeutic target for medulloblastoma. However, targeted therapy for ErbB signaling has remained a challenge because of its complex nature.

Cardiac glycosides, such as digoxin, digitoxin, and ouabain are naturally derived steroid-like compounds best appreciated for the treatment of congestive heart failure and as antiarrhythmic agents. Less well known is their emerging role as possible cancer therapeutics 11,12, although, the first generation of glycoside-based anticancer drugs are being tested in clinical trials. Retrospective clinical analyses revealed that administration of the cardiac glycoside digoxin during chemotherapy had a positive impact on patient survival in various cancers 13. Cardiotonic steroids are specific inhibitors of Na,K-ATPase, a ubiquitous membrane protein consisting of a catalytic *α*-subunit and a *β*-subunit, and an accessory third subunit belonging to the family of FXYD proteins with a more tissue-specific distribution 14,15. Using the energy derived from ATP hydrolysis, the enzyme pumps three Na^+^ out and two K^+^ in for each cycle thereby generating a Na^+^ gradient across the plasma membrane that is crucial for the normal functioning of epithelial and neuronal cells. In recent years, additional functions of Na,K-ATPase beyond ion transport have emerged and a role as signaling scaffold is now well accepted with ouabain-binding to Na,K-ATPase modulating an ever increasing array of signaling pathways in both pump-dependent and pump-independent manners 16. While Src is the best characterized pump-associated signaling molecule, Na,K-ATPase interacts with many other partners, including PI3K, caveolin-1, protein phosphatase 2, and EGFR 17–20. Many of these pathways have been linked to cancer and are involved in cell growth, apoptosis, cell adhesion, and cell motility. In addition, we and others have shown that Na,K-ATPase expression and function is affected in various human solid tumors 21–23. In fact, these differences might contribute to the selective effect of cardiac glycosides on tumor but not normal cells that has been observed in a variety of cancers 12. Nevertheless, sensitivity to cardiac glycosides on cell proliferation and viability might depend on tumor type and cardiac glycosides might not be good candidates as cancer therapeutics in all tumors 24. Interestingly, Na,K-ATPase inhibiting drugs have been shown to reduce the growth of the aggressive brain tumor glioblastoma 25. In glioblastoma, one of the most common found abnormalities is the overexpression or aberrant activation of EGFR or its constitutively active mutant EGFRvIII. In these tumors, the MAPK/Erk1/2 and the PI3K/Akt pathways have been identified as the driving forces of cellular proliferation and tumor progression. Akt activation is also frequently found in medulloblastoma and proliferation of medulloblastoma cells is dependent on PI3K/Akt signaling 26. ErbB2 overexpression has been shown to be one of the mechanisms involved in Akt activation in medulloblastoma cells 9. In the present study, we examined the effects of the cardiac glycoside ouabain on EGF-induced signaling in medulloblastoma cells. We show that while ouabain did not directly affect EGFR and ErbB2 phosphorylation, it inhibited EGF-induced activation of Erk1/2 and Akt signaling. In addition, we provide evidence that ouabain attenuated the formation of EGF-induced stress fibers and inhibited motility in medulloblastoma cells. Thus, we suggest that cardiac glycosides might prove beneficial for the treatment of the ErbB-positive aggressive forms of medulloblastoma.

## Materials and Methods

### Cell cultures

The human medulloblastoma cell line DAOY was obtained from the American Type Culture Collection (Rockville, MD). Cells were maintained in Dulbecco's Modified Eagle Medium/Nutrient Mixture F-12 (DMEM/F12) supplemented with 10% fetal bovine serum (FBS), 100 *μ*g/mL penicillin/streptomycin, 2 mmol/L l-glutamine, and nonessential amino acids (Invitrogen, Carlsbad, CA) at 37°C in a humidified 5% CO_2_ incubator. Experiments were performed on subconfluent monolayers after 4 h of serum starvation. Cells were pretreated for 4 h with 50 *μ*mol/L ouabain dissolved in ethanol as indicated. EGF was dissolved in ethanol since we consistently observed that dimethyl sulfoxide activated Erk1/2 signaling in DAOY cells. EGF was used at a final concentration of 10 ng/mL, unless noted otherwise, and insulin-like growth factor (IGF) at 100 ng/mL. The amount of ethanol used as vehicle in all experiments was kept at a constant and control cells were treated accordingly with the vehicle.

### Immunoblotting

Antibodies recognizing EGFR phosphorylated at tyrosine residues 1173, 1045, or 1068 were obtained from Cell Signaling Technology (Danvers, MA). Anti-EGFR antibody was purchased from Santa Cruz (Dallas, TX). Antibodies for phosphorylated, activated HER2, total HER2, phospho-Akt (Ser 473), phospho-Akt (Thr 308), total Akt, phospho-Raf (Ser 338), phosho-GSK3 beta (Ser 9), phospho-p38 (Thr 180, Tyr 182), phospho-JNK (Thr 183, Tyr 185), phospho-c-Jun (Ser 63), phospho-HSP27 (Ser 82), phospho-Erk1/2, total Erk1/2, and horseradish peroxidase (HRP)-conjugated secondary antibody were purchased from Cell Signaling Technology. Anti-PARP (Poly ADP ribose polymerase) antibody was purchased from BD Biosciences (San Jose, CA).

Cell lysates were prepared in a buffer containing 20 mmol/L Tris (pH 7.5), 150 mmol/L NaCl, 1 mmol/L EDTA, 1 mmol/L EGTA, 1% Triton X-100, 2.5 mmol/L sodium pyrophosphate, 1 mmol/L *β*-glycerophosphate, 1 mmol/L sodium vanadate, 1 mmol/L phenylmethylsulfonyl fluoride, and 5 *μ*g/mL of antipapain, leupeptin, and pepstatin (protease inhibitor cocktail). Cell lysates (20–40 *μ*g) were separated by 8% SDS-PAGE, transferred to nitrocellulose membranes and blocked in 5% nonfat milk in Tris-buffered saline with 0.1% Tween 20 (TBST). The blocked membranes were incubated overnight at 4°C with primary antibodies diluted in 5% nonfat milk/TBST (EGFR) or in 5% bovine serum albumin/TBST (p-Akt, Akt, p-Erk1/2, Erk1/2, p-EGFR, pHER2, HER2, pRAF, pGSK3, p-JNK, p-c-Jun, JNK, c-Jun, p-HSP27, HSP27, p-p38). Protein bands were detected with HRP-conjugated secondary antibodies in 5% nonfat milk/TBST and Enhanced Chemiluminescense Plus (GE Healthcare, Piscataway, NJ).

### Immunofluorescence and confocal microscopy

Antibody against the active, phosphorylated form of focal adhesion kinase (pFAK Y392) was purchased from BD Biosciences. Alexa 488™-conjugated secondary antibody and phalloidin-Alexa 546™ were obtained from Molecular Probes/Invitrogen (Carlsbad, CA). Cells grown on glass coverslips were fixed in 2% paraformaldehyde/phosphate buffered saline (PBS) followed by permeabilization in cold methanol (−20°C). Anti-pFAK (Y392) was diluted in PBS with 1% bovine serum albumin (PBS-BSA) and incubated overnight at 4°C followed by Alexa 488™-conjugated anti-mouse secondary antibody. Z-stacks were acquired sequentially for Alexa-488 and Alexa-546 dyes. In order to visualize actin stress fibers, five sections from each Z-stack were used to create maximum projections. Images were acquired with a Leica TCS SP5 laser-scanning confocal microscope and LSM software (Leica Microsystems, Mannheim, Germany). At least 100 cells per treatment group were screened for the presence of stress fibers. The ratio of cells relative to the total amount of cells analyzed for each group was determined from three independent experiments.

### Ras activation assay

DAOY cells grown on 100 mm dishes were serum-starved overnight, pretreated with 50 *μ*mol/L ouabain for 4 h followed by 10 ng/mL EGF for 15 min. Control cells were treated with an equal volume of solvent. The Ras activation assay was carried out following the manufacturer's instructions for the Ras Activation Assay Biochem Kit (Cytoskeleton, Inc., Denver, CO). Briefly, cell lysates were prepared using 400 *μ*L of lysis buffer, and then incubated with 20 *μ*L of Raf-RBD beads for 1 h at 4°C. The beads were washed once with 500 *μ*L of wash buffer. The samples were analyzed by 12% SDS-PAGE and the amount of activated Ras was determined by immunoblotting with a Ras pan-specific antibody.

### Cytotoxicity assay

Lactate dehydrogenase (LDH) levels were determined after 48 h of treatment using the Nonradioactive Cytotoxicity Kit (Promega, Madison, WI) according to manufacturer's instructions. Briefly, to obtain the released LDH, media were collected and cell debris was removed by a brief centrifugation. Viable cell LDH was collected after adding back 1 mL of fresh serum-free medium. Cells were lysed by freezing for 15 min at −70°C followed by thawing at 37°C. The medium was collected and cleared from cell debris by centrifugation. The relative release of LDH was determined as the ratio of released LDH versus total LDH from viable cells. Assays were performed twice in triplicate.

### Wound healing assay

DAOY cells were cultured until confluent and then a uniform cell-free area was created by scratching the monolayer with a plastic pipet tip. At 0 and 24 h of treatment with EGF or ouabain alone or in combination, four pictures each were taken and the distance between the two opposing edges was measured at two points. The distance migrated was calculated as difference of the scratch width at 0 h and that at 24 h. Data were normalized to vehicle-treated cells and represent the mean ± SD from three independent experiments.

## Results

### Activation of EGF signaling in DAOY medulloblastoma cells

**Figure 1:**
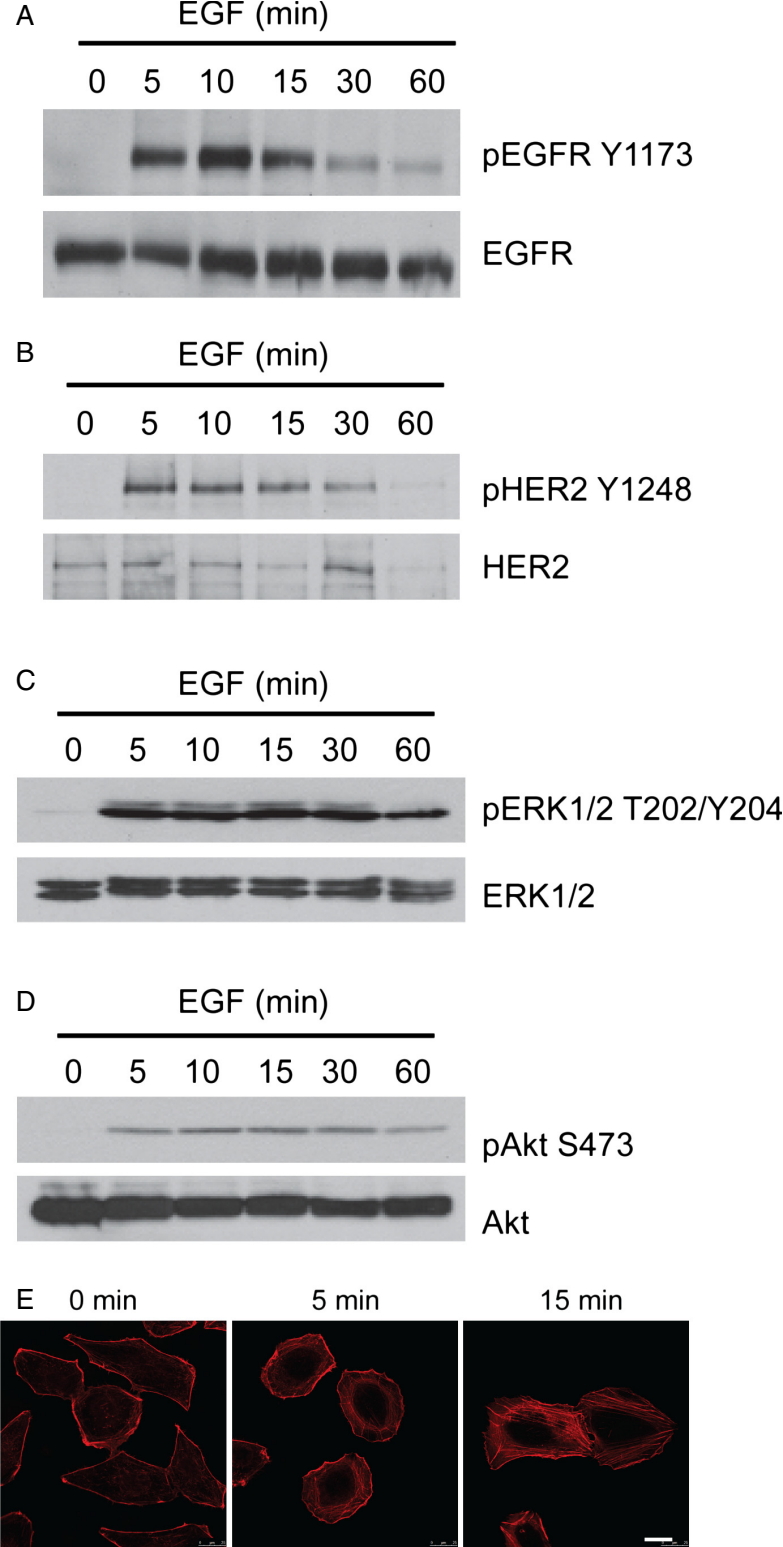
Activation of EGFR and HER2/ErbB2 signaling in DAOY cells. DAOY cells in serum-free medium were treated with 10 ng/mL EGF for the indicated times. Activation of EGFR (A) and HER2/ErbB2 (B) was determined by immunoblotting using antiphospho-EGFR (Tyr1173) and antiphospho-HER2 antibodies, respectively, which recognize only the activated form of the receptor. Immunoblots for total EGFR (A) and HER2 (B) confirm equal loading. Erk1/2 and Akt activation as determined by immunoblotting with phosphorylation-specific antibodies against Erk1/2 (T202/Y204) (C) and Akt (S473) (D), respectively. Equal loading was confirmed with antibodies for total Erk1/2 and Akt. (E) Phalloidin staining for filamentous actin in EGF-treated DAOY cells. Bar, 20 *μ*m. EGF, epidermal growth factor.

Human DAOY cells, often used as a model system for medulloblastoma in cell culture and tumor xenografts, express high levels of EGFR (Fig.1A) and HER2/ErbB2 (Fig.1B) 4. In contrast to the other EGFR/ErbB family members, HER2 is a ligand-less receptor but heterodimerizes with and transactivates other ErbB receptors 27. Upon EGF treatment, EGFR and HER2 were activated in DAOY cells as early as 5 min after addition of the ligand (Fig.1A and B). Receptor phosphorylation peaked around 10 min and then gradually decreased to levels similar to untreated cells after about 60 min of treatment. EGFR and HER2 activation induced a variety of signaling pathways, including the well-studied Erk1/2/MAPK and Akt signaling cascades. Phospho-Erk1/2 (Fig.1C) and phospho-Akt (Fig.1D) levels increased within 5 min of treatment with sustained activation up to 1 h and were accompanied by the induction of actin stress fibers (Fig.1E). Taken together, these data show that EGFR/HER2 signaling can be activated by EGF in DAOY cells and thus, DAOY cells may serve as a suitable model to test the effect of cardiac glycosides on EGFR signaling in medulloblastoma.

### Ouabain inhibits EGF signaling in medulloblastoma cells

**Figure 2:**
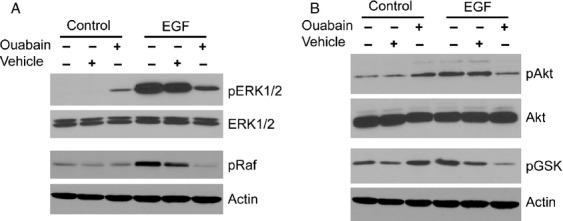
Ouabain inhibits EGF-induced Erk1/2 and Akt signaling. DAOY cells were incubated for 15 min with EGF in the presence or absence of 50 *μ*mol/L ouabain. (A) Activation of the MAPK signaling cascade was monitored by immunoblotting with phospho-specific anti-Erk1/2 and anti-Raf antibodies. Total Erk1/2 and actin levels confirm equal loading, respectively. (B) Activation of the Akt signaling cascade was determined with anti-phospho-Akt and anti-phospho-GSK antibodies that recognize only the activated forms of these proteins. Equal loading of cell lysate was confirmed with total Akt and actin immunoblots, respectively. EGF, epidermal growth factor.

Both cardiac glycosides (such as ouabain) and EGFR signaling can activate Erk1/2 and Akt. It has been suggested that binding of ouabain to Na,K-ATPase transactivates the EGFR through the nonreceptor tyrosine kinase Src to activate Erk1/2 and Akt signaling pathways (reviewed in 16) and indeed, ouabain treatment of DAOY cells increased Erk1/2 (Fig.2A) and Akt (Fig.2B) phosphorylation. Surprisingly, simultaneous treatment of DAOY cells with ouabain and EGF did not cause additive activation of the MAPK or Akt signaling pathways but rather resulted in reduced phosphorylation of the Erk1/2-Raf and Akt-GSK signaling axes, suggesting that ouabain inhibits ligand-activated EGFR signaling (Fig.2A and B). This effect appeared to be specific for EGF-induced signaling since ouabain did not inhibit IGF-1-induced signaling in these cells (Fig. S1). In addition, in D283 cells, a cell line derived from medulloblastoma metastasis in the peritoneum, ouabain did not inhibit EGF-induced activation of Erk1/2 but reduced EGF-induced Akt activation (Fig. S2A) and almost completely abolished EGF-induced EGFR phosphorylation (Fig. S2B). In U87 glioblastoma cells, ouabain did not inhibit EGF-induced Erk1/2 or Akt activation (Fig. S2A) and, surprisingly, further increased EGF-induced EGFR phosphorylation (Fig. S2B). Thus, it appears that the effects of ouabain on EGF-induced EGFR signaling are not due to nonspecific cytotoxicity but indeed due to inhibition of EGFR signaling.

**Figure 3:**
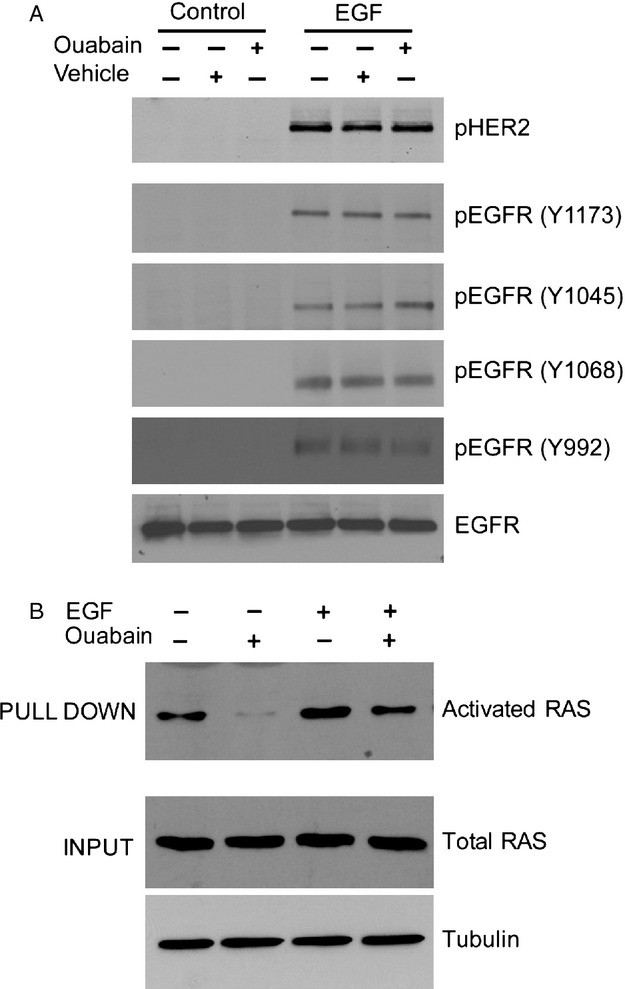
EGFR phosphorylation and Ras activity in ouabain-treated DAOY cells. (A) DAOY cells were incubated for 15 min with EGF in the presence of 50 *μ*mol/L ouabain. Activation of EGFR was monitored by immunoblotting with phospho-specific anti-EGFR antibodies recognizing different sites of phosphorylation. Total EGFR levels confirm equal loading. (B) Rac activity in DAOY cells treated with EGF in the presence of 50 *μ*mol/L ouabain was determined by a pulldown assay as described in Materials and Methods. *Upper panel*: immunoblot for activated Ras after pulldown; *middle panel*: immunoblot of total Ras to ensure equal input; *lower panel*: tubulin immunoblot as loading control. EGF, epidermal growth factor.

To test whether ouabain treatment of DAOY cells modulates EGFR itself, we monitored EGFR activation using a panel of antibodies recognizing specific phosphorylation sites of the receptor (pHER2, pEGFR[Y1173], pEGFR[Y1045], pEGFR[Y1068], pEGFR[Y992]). The various phosphorylated tyrosine residues of the receptor serve as binding sites for different secondary signaling molecules and define the specificity of the signaling pathway. However, while ouabain inhibited EGF-induced Erk1/2 and Akt signaling in DAOY cells, the HER2 or EGFR phosphorylation patterns induced by EGF treatment were not significantly altered by ouabain treatment (Fig.3). We also did not find significant changes in the overall level of tyrosine phosphorylated EGFR as determined by immunoprecipitation of the receptor followed by immunoblotting with antiphospho tyrosine antibody (data not shown). Although ouabain is thought to transactivate EGFR, ouabain itself did not induce HER2 or EGFR phosphorylation in DAOY cells (Fig.3A) suggesting that other factors downstream of the receptor might be involved in attenuating the EGFR signaling cascade in these cells. p21Ras is a signaling molecule common to both the Erk1/2 and Akt signaling pathways. Interestingly, in a Ras activation assay, ouabain not only inhibited EGF-induced Ras activation, but also reduced Ras activity in the absence of EGF. While the molecular connection remains to be determined our data suggest that ouabain could modulate EGFR signaling downstream of the receptor through inhibition of Ras.

### Activation of EGFR in the presence of ouabain induces stress signaling

**Figure 4:**
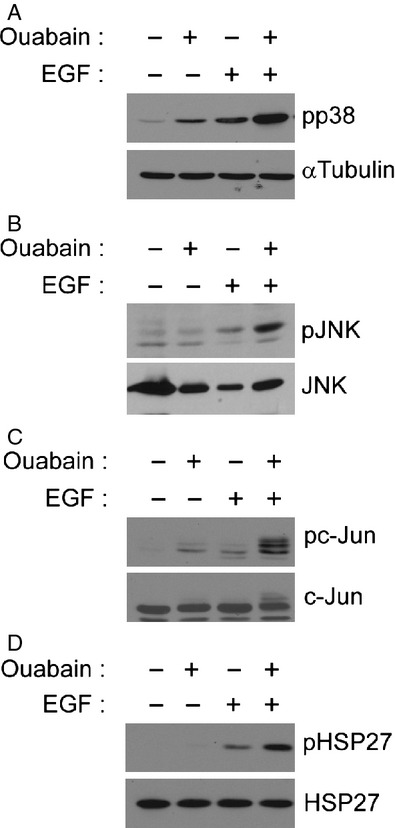
Simultaneous activation of EGF signaling and ouabain treatment induces stress signaling in DAOY cells. DAOY cells were incubated for 15 min with EGF in the presence of 50 *μ*mol/L ouabain. Activation of p38 MAPK (A), JNK (B), c-Jun (C), and HSP27 (D) were determined by immunoblotting with the respective phospho-specific antibodies. Total protein levels of each of the signaling molecules were determined to confirm equal loading. EGF, epidermal growth factor; HSPs, heat shock proteins.

Erk1/2 and Akt signaling are the best characterized targets of the EGF-induced signaling cascade, but EGF regulates many other pathways as well. Similarly, aside from Erk1/2 and Akt signaling, ouabain activates various other signaling pathways associated with the Na,K-ATPase signalosome 16. p38 MAPK, a member of the stress-activated MAPK family of serine/threonine kinases, can be activated by either EGF or ouabain 28,29. Treatment with ouabain or EGF individually increased the levels of phospho-p38 MAPK in DAOY cells (Fig.4A). However, and in contrast to Erk1/2 and Akt that were inhibited by simultaneous treatment with ouabain and EGF, the combined treatment of DAOY cells with EGF and ouabain resulted in increased activation of p38 MAPK beyond the levels observed by treatment with either EGF or ouabain alone (Fig.4A). The JNK signaling pathway is another signaling cascade commonly activated upon cellular stress. While ouabain alone did not activate JNK in DAOY cells, EGF treatment induced JNK phosphorylation and p-JNK levels were further increased upon combined treatment with EGF and ouabain (Fig.4B). Consistent with the activation of p38 MAPK and JNK signaling, either ouabain or EGF alone increased phosphorylation of the downstream signaling molecule c-Jun with much higher levels of phosphorylated c-Jun being observed in cells treated with EGF and ouabain simultaneously (Fig.4C). Together, these data suggest that activation of EGFR in the presence of ouabain induces a cellular stress response in medulloblastoma cells.

The cellular stress response is often associated with the induction of heat shock proteins (HSPs) followed by activation of either apoptotic cell death or cell survival mechanisms. In our experiments, simultaneous treatment of DAOY cells with EGF and ouabain did not induce cell death as determined by LDH release or induction of apoptosis markers such as PARP cleavage (data not shown) and thus, we screened for HSPs that have been implicated in cell survival. Hsp27 is a small HSP that has a protective role and can inhibit apoptosis by binding to and inactivating specific components of the apoptosis machinery 30. Treatment of DAOY cells with either ouabain or EGF alone or with ouabain and EGF in combination did not affect total HSP27 protein levels (Fig.4D). However, EGF treatment induced HSP27 phosphorylation which was further increased when both EGF and ouabain were present (Fig.4D). While HSP27 phosphorylation has been linked to cell survival and actin filament dynamics, the exact mechanisms are not well-defined. Nevertheless, our data imply that Hsp27 may be a possible candidate molecule mediating stress signaling in DAOY cells.

### Ouabain prevents EGF-induced actin reorganization and inhibits cell motility

**Figure 5:**
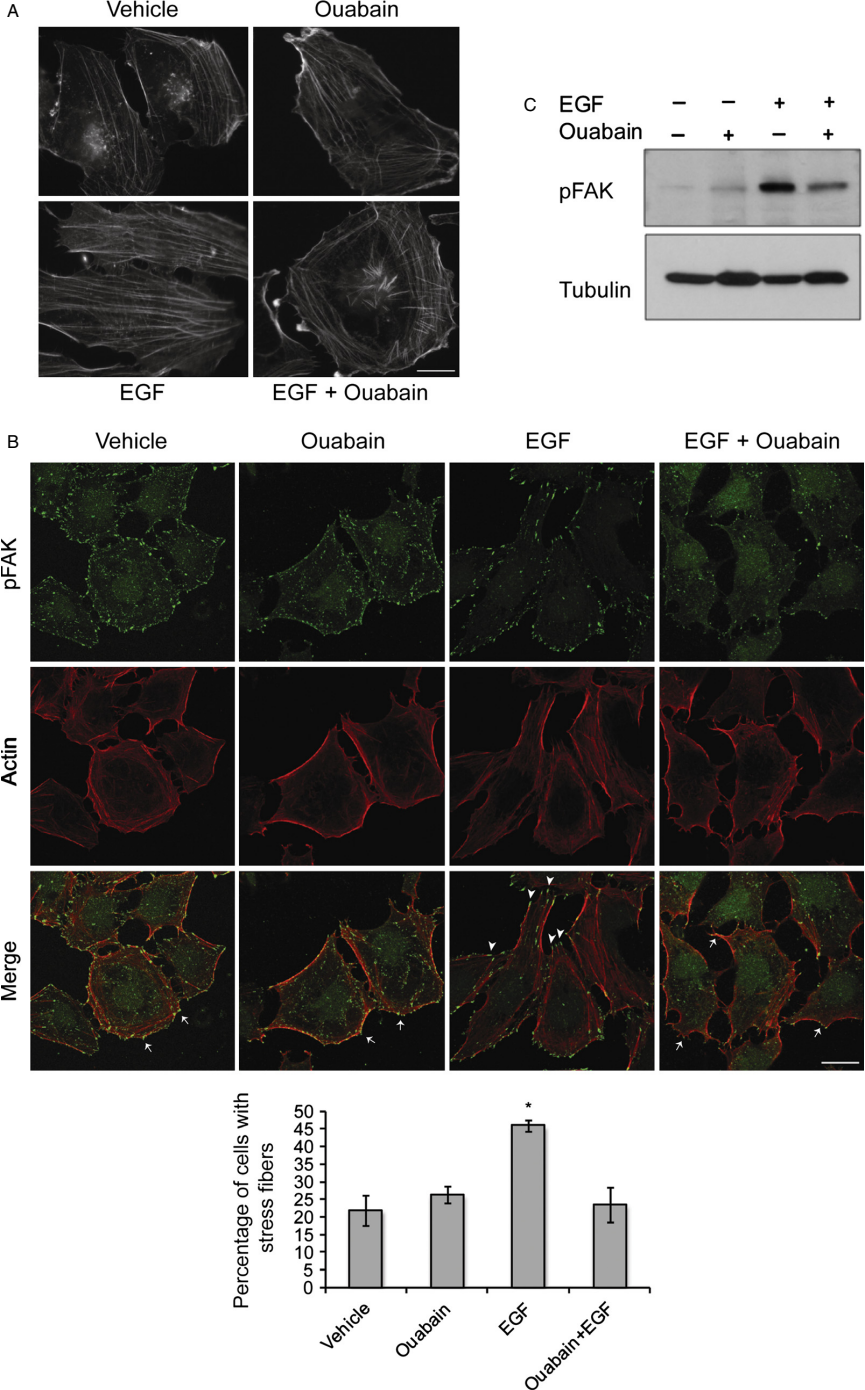
Ouabain prevents EGF-induced stress fiber formation. (A) Epifluorescence images of actin stress fibers in control cells and DAOY cells treated with ouabain, EGF, and EGF + ouabain for 2 h after FITC-conjugated phalloidin staining. Bar, 12.5 *μ*m. (B) Confocal images of activated, phosphorylated FAK (green), and actin stress fibers (red) in DAOY cells treated with and without EGF and ouabain as in (A). Arrows mark lamellipodia and arrowheads indicate phospho-FAK localized to the tips of actin stress fibers. Bar, 25 *μ*m. Graph shows quantitative data of number of cells with stress fibers relative to total number of cells counted per treatment group and represent average ± SE of three independent experiments, *P < 0.001. (C) DAOY cells were incubated with 10 ng/mL EGF in the presence or absence of 50 *μ*mol/L ouabain. Activation of FAK was monitored by immunoblotting with phospho-specific anti-FAK antibody. Tubulin immunoblot confirms equal loading. EGF, epidermal growth factor; FAK, focal adhesion kinase.

**Figure 6:**
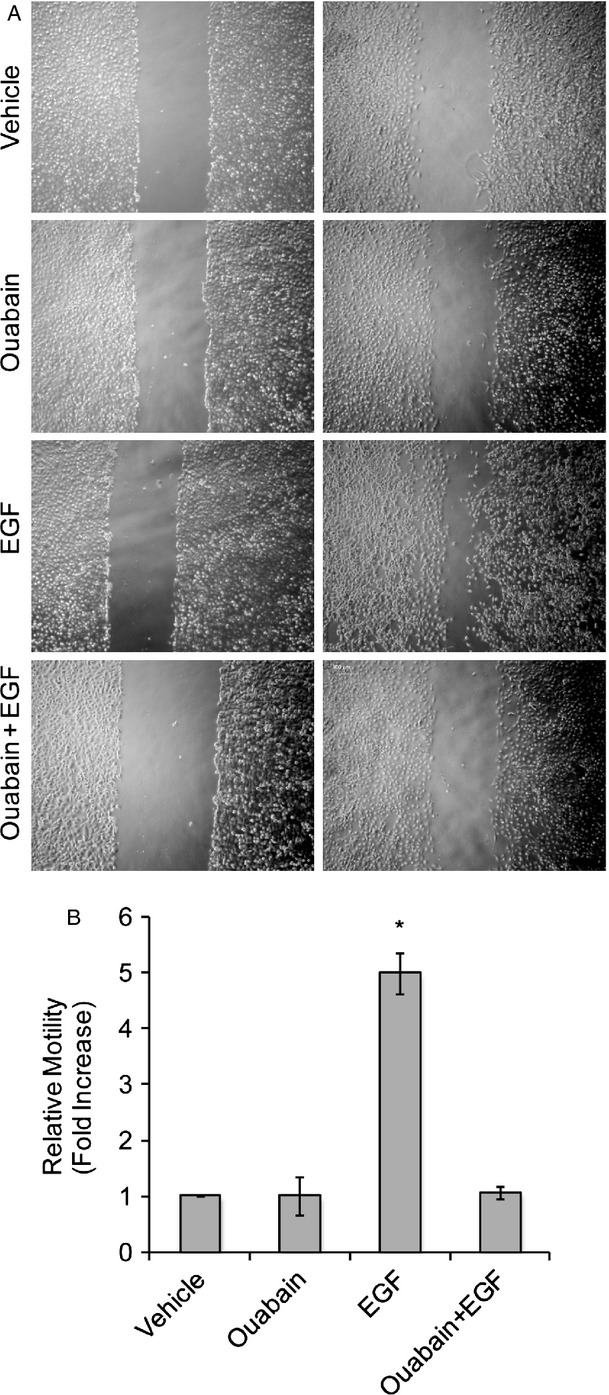
Ouabain inhibits EGF-induced cell motility. (A) Representative phase contrast images of a wound assay immediately after wounding (0 h) and after 24 h in control cells and in the presence of ouabain, EGF, or both. Bar, 500 *μ*m. (B) Quantitative analysis of cell migration in a wound assay. Data represent average ± SE of three independent assays done in triplicate, *P < 0.001. EGF, epidermal growth factor.

EGF is known to regulate cell migration in a variety of cell types, including cerebellar granule neurons 31 from which medulloblastoma is thought to derive. Consistent with a role in cell migration, EGF treatment of DAOY cells resulted in elongation of the cells and an increase in actin stress fibers when compared to vehicle-treated control cells (Fig.5A). The actin organization in ouabain-treated cells was comparable to that of control cells but ouabain treatment prevented the formation of actin stress fibers and the morphological changes induced by EGF. Focal adhesion kinase (FAK) has long been known as a regulator of cell migration and in many experimental systems enhanced FAK signaling promotes cell migration 32. Immunofluorescence staining revealed phospho-FAK localized to lamellipodia in control or ouabain-treated cells (Fig.5B, arrows), but in EGF-treated cells it was predominantly localized to the tips of actin stress fibers at the leading and trailing edges of the cells (Fig.5B, arrowheads). Nevertheless, in the presence of ouabain, EGF did not induce the characteristic phospho-FAK staining observed in DAOY cells treated with EGF alone, suggesting that ouabain might prevent EGF-induced cell migration in medulloblastoma cells. Similarly, while EGF treatment of DAOY cells resulted in increased FAK phosphorylation, ouabain treatment reduced EGF-induced FAK phosphorylation (Fig.5C) and prevented the formation of EGF-induced actin stress fibers (Fig.5B). Consistent with these results, we found that ouabain inhibited EGF-induced DAOY cell motility in a wound healing assay with ouabain and ouabain plus EGF-treated cells showing a similar migration pattern (Fig.6A and B). Together our results not only show that ouabain can inhibit EGF-induced oncogenic signaling, but also reduce EGF-induced cell migration, often a prerequisite for cancer cell metastasis.

## Discussion

Despite advances in treatment, medulloblastoma lags behind many other pediatric cancers in favorable outcome. One of the risk factors associated with poor survival in medulloblastoma is the aberrant expression of ErbB2/HER2 2. However, while the EGFR signaling cascade is an attractive therapeutic approach 3–5, targeted therapy for ErbB signaling has remained a challenge due to its complex nature. In recent years, Na,K-ATPase has been invoked in transactivating EGFR signaling, thereby activating its downstream effectors Erk1/2 and Akt 16. Nevertheless, cardiac glycosides such as digoxin, digitoxin, and ouabain have also been proposed as anticancer therapeutics. Thus, we thought to investigate the relationship between Na,K-ATPase and EGFR signaling in medulloblastoma cells. Here, we show that consistent with previous studies, treatment of DAOY medulloblastoma cells with EGF activated the EGFR signaling cascade and resulted in increased phosphorylation of both the EGFR and the HER2/ErbB2 receptors as well as the downstream targets Erk1/2 and Akt. Furthermore, ouabain appeared to activate Erk1/2 and Akt signaling independent of EGFR. However, ouabain inhibited EGF-induced activation of the Erk1/2 and Akt signaling pathways as well as p21 Ras activation, prevented EGF-induced formation of actin stress fibers, and inhibited EGF-induced FAK phosphorylation and DAOY cell motility, possibly through activation of stress signaling. Thus, these studies not only reveal a previously unknown crosstalk between Na,K-ATPase and EGFR signaling, but also suggest that cardiac glycosides might be effective as anticancer therapeutics especially in the more aggressive and invasive cancers associated with aberrant activation of the EGFR signaling cascade.

### Na,K-ATPase as a target in brain tumors

Increasing evidence suggests that ion channels and pumps not only function in regulating membrane potential, ion homeostasis, and electric signaling in excitable cells, but also play additional important roles in regulating cell proliferation, migration, apoptosis, and differentiation. At least in part, these roles may be independent of ion flow, as such functions have traditionally been assigned to be regulated by intracellular signaling proteins. Consistent with such a role in cell signaling, channel proteins and ion pumps have been reported to form macromolecular complexes with growth factors, cell adhesion molecules, and other signaling molecules. As these nontraditional roles of ion pumps and channels are being recognized, it is increasingly being suggested that these proteins might contribute to cancer progression 33. Indeed in recent years, aberrant expression and/or function of several classes of ion channels have been found in various cancers including brain tumors 34. One of the best characterized brain tumors in this regard is glioblastoma, in which changes in sodium, potassium, chloride, and calcium channels have been suggested to contribute to the malignant behavior. For example, big conductance K^+^ (BK) channels are overexpressed in malignant gliomas and correlate positively with the malignancy grade of tumors. A novel splice isoform, gBK, has exclusively been observed in glioma and may contribute to invasive cell migration in this cancer 35. Aberrant expression of the ether à go-go K^+^ channel 1 (EAG1) has been reported in more than three quarters of human tumors, and EAG1 and the EAG-related channel 1 (ERG1) have been found to be upregulated in glioblastoma 35. While the contribution of ion channels toward medulloblastoma tumorigenesis and progression are very poorly explored, a recent study reported EAG2, that shares similar electrophysiological properties with EAG1, to be overexpressed in a significant subset of mouse and human medulloblastoma across molecular (WNT, SHH, or Group 4) and histological (nodular, classic, desmoplastic, or anaplastic) subgroups and to control mitotic entry and tumor growth 36. Another important candidate channel in brain tumor pathogenesis is the ClC-3 chloride channel which has been suggested to facilitate migrating behavior in glioblastoma cells 34 and inhibition of ClC-3 channels with chlorotoxin markedly inhibited glioblastoma cell invasion in vitro 37. Other studies found that chlorotoxin, a 4-kD peptide purified from *Leiurus quinquestriatus* scorpion and inhibitor of small chloride channels is a highly specific marker for gliomas and tumors of neuroectodermal origin including medulloblastoma 38. Thus, in recent years, significant effort has been made to translate these findings into clinical applications including drug targeting 39 and in vivo bio-imaging 40.

Changes in Na,K-ATPase expression and function are now well documented in various human solid tumors 21,41,42, and have been suggested to contribute to the selective effect of cardiac glycosides in at least some of these cancers 12. In human glioblastoma, the *α*_1_-subunit of Na,K-ATPase is highly expressed, and inhibition of the *α*_1_-subunit impaired cell migration, cell proliferation, and increased the in vivo survival of mice with orthotopic tumor xenografts 25,43. Studies on Na,K-ATPase in medulloblastoma are still lagging behind. Although recently overexpression of both the *α*_1_ and the *α*_3_ isoforms was reported in such tumors, and thus Na,K-ATPase might be a valid therapeutic target for medulloblastoma 42. Our studies show that the cardiac glycoside and specific inhibitor of Na,K-ATPase, ouabain, can inhibit EGF-induced signaling and cell migration in medulloblastoma cells, but not glioblastoma cells, and indeed support the notion of developing novel therapeutic approaches targeting the sodium pump. Nevertheless, a recent study showed that normal breast tumor cells were more sensitive to the cytotoxic effects of cardiac glycosides than human breast tumor cells 24. Since cardiac glycosides are toxic to some degree to all cells, it remains to be determined whether the Na,K-ATPase expression levels and/or isoform patterns could confer therapeutic advantage and whether the EGFR activation status has to be considered for therapeutic decision making. In addition, previous reports of Src activation in response to ouabain suggest that cardiac glycosides could enable cell survival and proliferation 44. Src has been shown to directly interact with the *α*-subunit of Na,K-ATPase and has been invoked in the cardiac glycoside-induced activation of Erk1/2 and Akt signaling 45. Nevertheless, under our experimental conditions we did not find Src to be associated with Na,K-ATPase in DAOY cells (data not shown). Thus, it remains to be determined whether in DAOY cells other mechanisms are involved in ouabain-induced Erk1/2 and Akt activation, such as an increase in intracellular calcium or direct association with PI3K, as has been shown in other cell systems 46.

### EGF-induced neuronal migration requires a functional Na,K-ATPase

In epithelial cells, it is well established that EGFR activation is involved in cell migration. However, studies on EGFR signaling in neuronal or neuronal progenitor cells are limited. In the CNS, EGFR is expressed in glial cells and neurons of the hippocampus, cerebellum, and cerebral cortex. In EGFR knockout mice, ectopic neurons were detected in the white matter of the hippocampus suggesting that EGFR signaling may be involved in neuronal migration 47. In glioblastoma multiforme, it is well established that overexpression and mutation of the EGFR contributes to aggressiveness through increased proliferation, survival, and migration. In neural stem cells, EGFR signaling conferred a motile phenotype and blocked neuronal differentiation 48,49. ErbB2 has been suggested to be involved in the migration of medulloblastoma cells 2,9,10, and in our studies DAOY cells treated with EGF were highly motile, suggesting that activation of the EGFR signaling cascade may play a role in medulloblastoma cell migration.

In DAOY cells, EGF-induced cell motility was accompanied by a remodeling of the actin cytoskeleton with a diminished cortical actin ring and the formation of stress fibers which are usually found in highly motile cells. Nevertheless, ouabain not only inhibited EGF-induced Erk1/2 and Akt signaling, but also reduced EGF-induced cell motility, and prevented the morphological changes in the actin cytoskeleton that were induced by EGF. These results are indeed consistent with our previous reports in epithelial cells in which Na,K-ATPase inhibition by ouabain prevented the transient formation of actin stress fibers during epithelial polarization 50. In epithelial cells, ouabain treatment also inhibited the activity of RhoA GTPase 50, a member of the Rho family of small GTP-binding proteins which has been implicated in the regulation of actin stress fiber formation. It is well known that Rho GTPases modulate the activity of other GTPases. Mutual inhibition and positive feedback of each other have been described for both RhoA and Rac1 51. Interestingly, recent studies showed that Rac1 is overexpressed in medulloblastoma tumors and that Rac1 depletion strongly inhibits medulloblastoma cell invasion 52. We previously showed that the expression of the *β*_1_-subunit of Na,K-ATPase in transformed cells resulted in activation of Rac1 and suppression of cell motility 17. While it remains to be determined, it is possible that in medulloblastoma cells as well, loss of Na,K-ATPase function could modulate RhoA or Rac1 activity to inhibit EGF-induced motility and stress fiber formation.

The rearrangement in the actin cytoskeleton in EGF-treated DAOY cells was accompanied by a relocation of the active, phosphorylated form of FAK to stress fiber endpoints. With its role in cell migration, relocation of FAK to stress fiber endpoints in EGF-treated cells supports the hypothesis that the EGFR signaling cascade might be involved in switching from random to directional movement, thus promoting tumor cell invasion in medulloblastoma cells. As FAK functions in linking integrins and growth factor signaling to cell adhesion, invasion, proliferation, and migration 32, it has emerged as a key player in the progression of many cancers. FAK expression and/or phosphorylation have been correlated with malignancy, metastatic disease, and poor patient prognosis in various cancers, including glioma 53. In medulloblastoma, a recent study reported that c-Met inhibition reduced cell migration and cell invasion in vitro and in tumor xenografts. These effects were more pronounced when the c-Met inhibitor was combined with a small-molecule FAK inhibitor, suggesting that simultaneous administration of c-Met and FAK inhibitors could constitute a new potential strategy for medulloblastoma therapy 54. In our studies, treatment of DAOY cells with ouabain prevented EGF-induced relocation of phosphorylated FAK. Thus, we suggest that simultaneous targeting of Na,K-ATPase, EGFR, and possibly FAK signaling might as well confer therapeutic advantage in medulloblastoma.

## Conclusions

Aberrant activation of the EGFR signaling cascade has been associated with poor prognosis in medulloblastoma. Nevertheless, in a phase II study in children with refractory CNS malignancies lapatinib did not inhibit target in tumor and had little single agent activity 55. Thus, understanding the molecular biology of medulloblastoma remains a prerequisite to develop more targeted and effective therapeutics. In medulloblastoma, ErbB2 overexpression upregulates prometastatic genes, increases the migration of medulloblastoma cells, and has been associated with advanced metastatic disease and poor clinical outcome 2,9,10. We now show that the cardiac glycoside ouabain, an inhibitor of Na,K-ATPase, inhibits EGFR signaling in medulloblastoma cells, but not glioblastoma cells, and prevents EGF-induced cell migration, a prerequisite for an invasive phenotype. While cardiac glycosides might not prove useful in all tumor types as anticancer therapeutics, we suggest that Na,K-ATPase is an attractive target to develop novel therapeutic strategies for medulloblastoma with aberrant activation of EGFR signaling. Not only does Na,K-ATPase modulate signaling molecules that are thought to be central to medulloblastoma pathogenesis, but also due to its plasma membrane localization and therefore accessibility from the extracellular side it makes a very attractive pharmaceutical target.

## Supporting Information

**Figure S1.** Activation of IGF-induced signaling in ouabain-treated cells. DAOY cells were incubated for 30 min with 100 ng/mL IGF-1 in the presence or absence of 50 *μ*mol/L ouabain. Activation of the IGF-1 receptor (IGF1R) was determined by immunoblotting with phospho-specific IGF-1R antibody. Activation of the MAPK and Akt signaling cascades was monitored by immunoblotting with phospho-specific anti-Erk1/2 and anti-Akt antibodies as described for EGF experiments. Total IGF1R, Erk1/2, Akt, and tubulin levels confirm equal loading.

**Figure S2.** Effects of ouabain on EGF-induced signaling. D283 cells (A, B), U87 glioblastoma cells (A, B), and HEK293T human kidney epithelial cells (C) were incubated for 15 min with EGF in the presence or absence of 50 *μ*mol/L ouabain. (A, C) Activation of the MAPK and Akt signaling cascade was monitored by immunoblotting with phospho-specific antibodies. Equal loading of cell lysate was confirmed with total MAPK and Akt immunoblots. (B) Activation of EGFR was monitored by immunoblotting with the antiphospho-EGFR (Tyr1173) antibody. Total EGFR levels confirm equal loading.

## References

[1] Northcott PA, Jones DT, Kool M, Robinson GW, Gilbertson RJ, Cho YJ (2012). Medulloblastomics: the end of the beginning. Nat. Rev. Cancer.

[2] Gilbertson RJ, Perry RH, Kelly PJ, Pearson AD, Lunec J (1997). Prognostic significance of HER2 and HER4 coexpression in childhood medulloblastoma. Cancer Res.

[3] Abouantoun TJ, MacDonald TJ (2009). Imatinib blocks migration and invasion of medulloblastoma cells by concurrently inhibiting activation of platelet-derived growth factor receptor and transactivation of epidermal growth factor receptor. Mol. Cancer Ther.

[4] Meco D, Servidei T, Riccardi A, Ferlini C, Cusano G, Zannoni GF (2009). Antitumor effect in medulloblastoma cells by gefitinib: ectopic HER2 overexpression enhances gefitinib effects in vivo. Neuro. Oncol.

[5] Meco D, Servidei T, Zannoni GF, Martinelli E, Prisco MG, de Waure C (2010). Dual inhibitor AEE788 reduces tumor growth in preclinical models of medulloblastoma. Transl. Oncol.

[6] Wong RW, Guillaud L (2004). The role of epidermal growth factor and its receptors in mammalian CNS. Cytokine Growth Factor Rev.

[7] Yamada M, Ikeuchi T, Hatanaka H (1997). The neurotrophic action and signalling of epidermal growth factor. Prog. Neurobiol.

[8] Povlsen GK, Berezin V, Bock E (2008). Neural cell adhesion molecule-180-mediated homophilic binding induces epidermal growth factor receptor (EGFR) down-regulation and uncouples the inhibitory function of EGFR in neurite outgrowth. J. Neurochem.

[9] Hernan R, Fasheh R, Calabrese C, Frank AJ, Maclean KH, Allard D (2003). ERBB2 up-regulates S100A4 and several other prometastatic genes in medulloblastoma. Cancer Res.

[10] Gilbertson R, Wickramasinghe C, Hernan R, Balaji V, Hunt D, Jones-Wallace D (2001). Clinical and molecular stratification of disease risk in medulloblastoma. Br. J. Cancer.

[11] Prassas I, Diamandis EP (2008). Novel therapeutic applications of cardiac glycosides. Nat. Rev. Drug Discov.

[12] Newman RA, Yang P, Pawlus AD, Block KI (2008). Cardiac glycosides as novel cancer therapeutic agents. Mol. Interv.

[13] Menger L, Vacchelli E, Adjemian S, Martins I, Ma Y, Shen S (2012). Cardiac glycosides exert anticancer effects by inducing immunogenic cell death. Sci. Trans. Med.

[14] Kaplan JH (2002). Biochemistry of Na,K-ATPase. Annu. Rev. Biochem.

[15] Lingrel JB (2010). The physiological significance of the cardiotonic steroid/ouabain-binding site of the Na,K-ATPase. Annu. Rev. Physiol.

[16] Reinhard L, Tidow H, Clausen M, Nissen P (2013). Na+, K+-ATPase as a docking station: protein–protein complexes of the Na+, K+-ATPase. Cell. Mol. Life Sci.

[17] Barwe SP, Anilkumar G, Moon SY, Zheng Y, Whitelegge JP, Rajasekaran SA (2005). Novel role for Na,K-ATPase in phosphatidylinositol 3-kinase signaling and suppression of cell motility. Mol. Biol. Cell.

[18] Cai T, Wang H, Chen Y, Liu L, Gunning WT, Quintas LE (2008). Regulation of caveolin-1 membrane trafficking by the Na/K-ATPase. J. Cell Biol.

[19] Haas M, Wang H, Tian J, Xie Z (2002). SRC-mediated inter-receptor cross-talk between the Na+/K+-ATPase and the epidermal growth factor receptor relays the signal from ouabain to mitogen-activated protein kinases. J. Biol. Chem.

[20] Kimura T, Han W, Pagel P, Nairn AC, Caplan MJ (2011). Protein phosphatase 2A interacts with the Na,K-ATPase and modulates its trafficking by inhibition of its association with arrestin. PLoS One.

[21] Mijatovic T, Van Quaquebeke E, Delest B, Debeir O, Darro F, Kiss R (2007). Cardiotonic steroids on the road to anti-cancer therapy. Biochim. Biophys. Acta.

[22] Espineda C C, Seligson DB, James Ball W, Rao J, Palotie A, Horvath S (2003). Analysis of the Na,K-ATPase alpha- and beta-subunit expression profiles of bladder cancer using tissue microarrays. Cancer.

[23] Rajasekaran SA, Ball WJ, Bander NH, Liu H, Pardee JD, Rajasekaran AK (1999). Reduced expression of beta-subunit of Na,K-ATPase in human clear-cell renal cell carcinoma. J. Urol.

[24] Clifford RJ, Kaplan JH (2013). Human breast tumor cells are more resistant to cardiac glycoside toxicity than non-tumorigenic breast cells. PLoS One.

[25] Lefranc F, Mijatovic T, Kondo Y, Sauvage S, Roland I, Debeir O (2008). Targeting the alpha 1 subunit of the sodium pump to combat glioblastoma cells. Neurosurgery.

[26] Hartmann W, Digon-Sontgerath B, Koch A, Waha A, Endl E, Dani I (2006). Phosphatidylinositol 3′-kinase/AKT signaling is activated in medulloblastoma cell proliferation and is associated with reduced expression of PTEN. Clin. Cancer Res.

[27] Ogimoto G, Yudowski GA, Barker CJ, Kohler M, Katz AI, Feraille E (2000). G protein-coupled receptors regulate Na+, K+-ATPase activity and endocytosis by modulating the recruitment of adaptor protein 2 and clathrin. Proc. Natl. Acad. Sci. USA.

[28] Li S, Wattenberg EV (1998). Differential activation of mitogen-activated protein kinases by palytoxin and ouabain, two ligands for the Na+, K+-ATPase. Toxicol. Appl. Pharmacol.

[29] Yarden Y, Sliwkowski MX (2001). Untangling the ErbB signalling network. Nat. Rev. Mol. Cell Biol.

[30] Mymrikov EV, Seit-Nebi AS, Gusev NB (2011). Large potentials of small heat shock proteins. Physiol. Rev.

[31] Martinez R, Eller C, Viana NB, Gomes FC (2011). Thyroid hormone induces cerebellar neuronal migration and Bergmann glia differentiation through epidermal growth factor/mitogen-activated protein kinase pathway. Eur. J. Neurosci.

[32] Schaller MD (2010). Cellular functions of FAK kinases: insight into molecular mechanisms and novel functions. J. Cell Sci.

[33] Kunzelmann K (2005). Ion channels and cancer. J. Membr. Biol.

[34] Sontheimer H (2008). An unexpected role for ion channels in brain tumor metastasis. Exp. Biol. Med. (Maywood).

[35] Molenaar RJ (2011). Ion channels in glioblastoma. ISRN Neurol.

[36] Huang X, Dubuc AM, Hashizume R, Berg J, He Y, Wang J (2012). Voltage-gated potassium channel EAG2 controls mitotic entry and tumor growth in medulloblastoma via regulating cell volume dynamics. Genes Dev.

[37] Soroceanu L, Manning TJ, Sontheimer H (1999). Modulation of glioma cell migration and invasion using Cl(−) and K(+) ion channel blockers. J. Neurosci.

[38] Lyons SA, O'Neal J, Sontheimer H (2002). Chlorotoxin, a scorpion-derived peptide, specifically binds to gliomas and tumors of neuroectodermal origin. Glia.

[39] Fu Y, An N, Li K, Zheng Y, Liang A (2012). Chlorotoxin-conjugated nanoparticles as potential glioma-targeted drugs. J. Neurooncol.

[40] Stroud MR, Hansen SJ, Olson JM (2011). In vivo bio-imaging using chlorotoxin-based conjugates. Curr. Pharm. Des.

[41] Rajasekaran SA, Rajasekaran AK (2009). Na,K-ATPase and epithelial tight junctions. Front. Biosci.

[42] Sunol M, Cusi V, Cruz O, Kiss R, Lefranc F (2011). Immunohistochemical Analyses of {alpha}1 and {alpha}3 Na+/K+-ATPase Subunit Expression in Medulloblastomas. Anticancer Res.

[43] Lefranc F, Kiss R (2008). The sodium pump alpha1 subunit as a potential target to combat apoptosis-resistant glioblastomas. Neoplasia.

[44] Kometiani P, Liu L, Askari A (2005). Digitalis-induced signaling by Na+/K+-ATPase in human breast cancer cells. Mol. Pharmacol.

[45] Tian J, Cai T, Yuan Z, Wang H, Liu L, Haas M (2006). Binding of SRC to Na+/K+-ATPase forms a functional signaling complex. Mol. Biol. Cell.

[46] Wu J, Akkuratov EE, Bai Y, Gaskill CM, Askari A, Liu L (2013). Cell signaling associated with Na(+)/K(+)-ATPase: activation of phosphatidylinositide 3-kinase IA/Akt by ouabain is independent of Src. Biochemistry.

[47] Sibilia M, Steinbach JP, Stingl L, Aguzzi A, Wagner EF (1998). A strain-independent postnatal neurodegeneration in mice lacking the EGF receptor. EMBO J.

[48] Ayuso-Sacido A, Moliterno JA, Kratovac S, Kapoor GS, O'Rourke DM, Holland EC (2010). Activated EGFR signaling increases proliferation, survival, and migration and blocks neuronal differentiation in post-natal neural stem cells. J. Neurooncol.

[49] Boockvar JA, Kapitonov D, Kapoor G, Schouten J, Counelis GJ, Bogler O (2003). Constitutive EGFR signaling confers a motile phenotype to neural stem cells. Mol. Cell. Neurosci.

[50] Rajasekaran SA, Palmer LG, Moon SY, Peralta Soler A, Apodaca GL, Harper JF (2001). Na,K-ATPase activity is required for formation of tight junctions, desmosomes, and induction of polarity in epithelial cells. Mol. Biol. Cell.

[51] Guilluy C, Garcia-Mata R, Burridge K (2011). Rho protein crosstalk: another social network?. Trends Cell Biol.

[52] Chen B, Gao Y, Jiang T, Ding J, Zeng Y, Xu R (2011). Inhibition of tumor cell migration and invasion through knockdown of Rac1 expression in medulloblastoma cells. Cell. Mol. Neurobiol.

[53] Ding L, Sun X, You Y, Liu N, Fu Z (2010). Expression of focal adhesion kinase and phosphorylated focal adhesion kinase in human gliomas is associated with unfavorable overall survival. Transl. Res.

[54] Guessous F, Yang Y, Johnson E, Marcinkiewicz L, Smith M, Zhang Y (2012). Cooperation between c-Met and focal adhesion kinase family members in medulloblastoma and implications for therapy. Mol. Cancer Ther.

[55] Fouladi M, Stewart CF, Blaney SM, Onar-Thomas A, Schaiquevich P, Packer RJ (2013). A molecular biology and phase II trial of lapatinib in children with refractory CNS malignancies: a pediatric brain tumor consortium study. J. Neurooncol.

