# Supplementary figures and images for: Inhibition of epidermal growth factor signaling by the cardiac glycoside ouabain in medulloblastoma
Source: Cancer Med. 2014 Jul 23;3(5):1146–58. doi: 10.1002/cam4.314 (PMC4302666; doi:10.1002/cam4.314)

Supplementary Figure S1

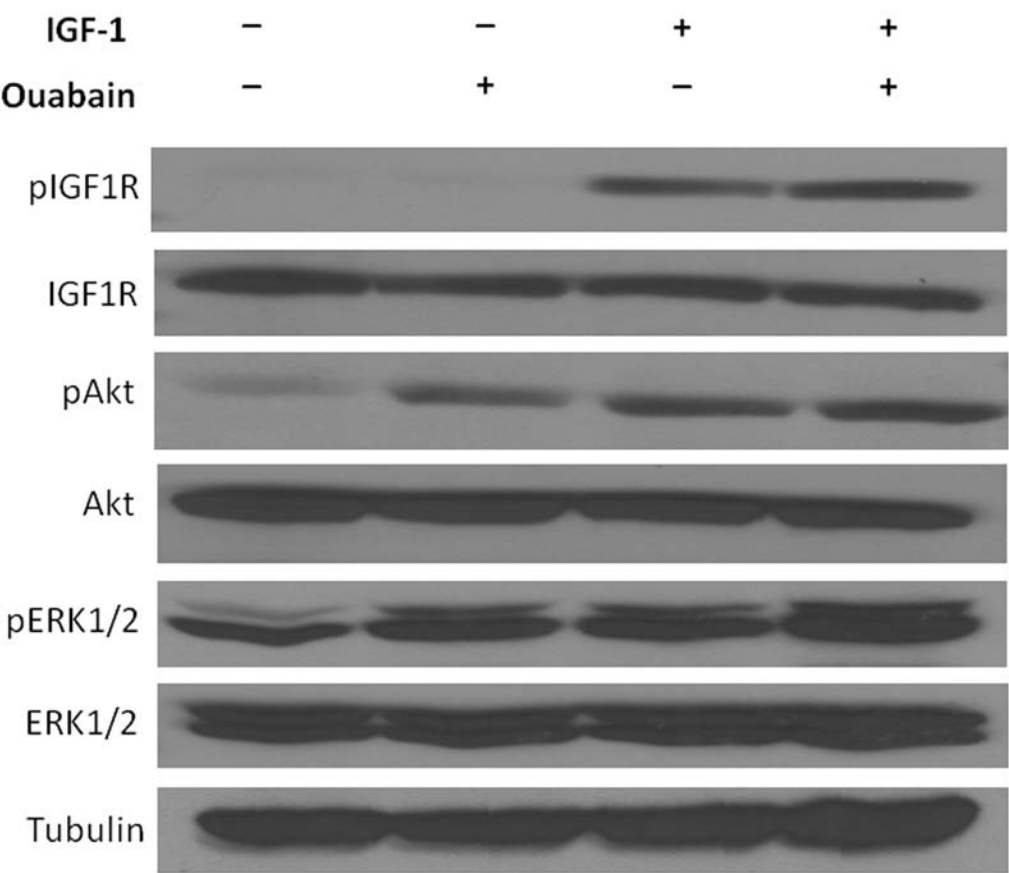

Supplement: Supplementary file 1 — Figure S1. Activation of IGF-induced signaling in ouabain-treated cells. DAOY cells were incubated for 30 min with 100 ng/mL IGF-1 in the presence or absence of 50 μmol/L ouabain. Activation of the IGF-1 receptor (IGF1R) was determined by immunoblotting with phospho-specific IGF-1R antibody. Activation of the MAPK and Akt signaling cascades was monitored by immunoblotting with phospho-specific anti-Erk1/2 and anti-Akt antibodies as described for EGF experiments. Total IGF1R, Erk1/2, Akt, and tubulin levels confirm equal loading. [file cam40003-1146-SD1.pdf]

Supplementary Figure S2

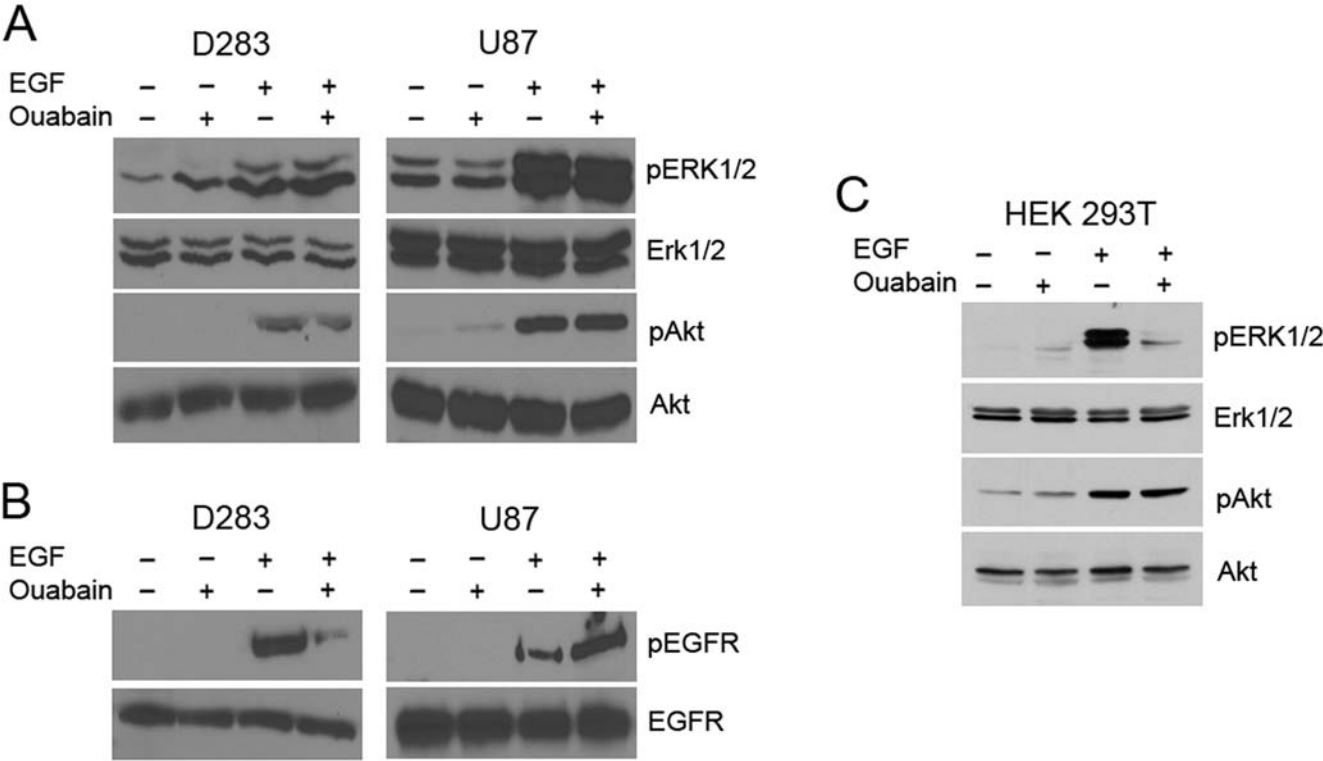

Supplement: Supplementary file 2 — Figure S2. Effects of ouabain on EGF-induced signaling. D283 cells (A, B), U87 glioblastoma cells (A, B), and HEK293T human kidney epithelial cells (C) were incubated for 15 min with EGF in the presence or absence of 50 μmol/L ouabain. (A, C) Activation of the MAPK and Akt signaling cascade was monitored by immunoblotting with phospho-specific antibodies. Equal loading of cell lysate was confirmed with total MAPK and Akt immunoblots. (B) Activation of EGFR was monitored by immunoblotting with the antiphospho-EGFR (Tyr1173) antibody. Total EGFR levels confirm equal loading. [file cam40003-1146-SD2.pdf]
